# Supplementary material for: Ominoxanthone—The First Xanthone Linearly Fused to a γ-Lactone from Cortinarius ominosus Bidaud Basidiomata. CASE- and DFT-Based Structure Elucidation
Source: Molecules. 2023 Feb 6;28(4):1557. doi: 10.3390/molecules28041557 (PMC9965508; doi:10.3390/molecules28041557)
Supplement: Supplementary file 1 [file molecules-28-01557-s001.zip › molecules-2181185-supplementary.pdf]

Ominoxanthone—The First Xanthone Linearly Fused to a γ-Lactone from *Cortinarius ominosus* Bidaud Basidiomata. CASE- and DFT-Based Structure Elucidation

Alice Trac [1], Célia Issaad [1], Mehdi A. Beniddir [1], Jean-Michel Bellanger [2], Jean-François Gallard [3], Alexei V. Buevich [4], Mikhail E. Elyashberg [5,*] and Pierre Le Pogam [1,*]

1 Équipe "Chimie des Substances Naturelles" BioCIS, CNRS, Université Paris-Saclay, 17 Avenue des Sciences, 91400 Orsay, France; alice.trac@universite-paris-saclay.fr (A.T.); celia.issd@gmail.com (C.I.); mehdi.beniddir@universite-paris-saclay.fr (M.A.B.)
2 CEFE, CNRS, Université Montpellier, EPHE, IRD, INSERM, 1919 Route de Mende, CEDEX 5, 34293 Montpellier, France; jean-michel.bellanger@cefe.cnrs.fr
3 Institut de Chimie des Substances Naturelles, CNRS, ICSN UPR 2301, Université Paris-Saclay, 91198 Gif-sur-Yvette, France; jean-francois.gallard@cnrs.fr
4 Process and Analytical Chemistry, Merck & Co., Inc., 2015 Galloping Hill Road, Kenilworth, New Jersey, NJ 07033, USA; alexei.buevich@merck.com
5 Advanced Chemistry Development Inc. (ACD/Labs), 8 King Street, Toronto, ON M5C 1B5, Canada
* Correspondence: mikhail.elyashberg@acdlabs.com (M.E.E.); pierre.le-pogam-alluard@universite-paris-saclay.fr (P.L.P.)

Table of Contents

N°	Content	Page
S1	UHPLC-DAD-MS^2 analysis of **1**.	S-3
S2	IR spectrum of **1**.	S-3
S3	^{1}H NMR (TFA-*d*, 700 MHz) spectrum of **1**.	S-4
S4	^{13}C NMR (TFA-*d*, 175 MHz) spectrum of **1**.	S-5
S5	COSY NMR (TFA-*d*, 700 MHz) spectrum of **1**.	S-6
S6	Edited HSQC NMR (TFA-*d*, 700/175 MHz) spectrum of **1**.	S-7
S7	HMBC NMR (TFA-*d*, 700/175 MHz) spectrum of **1**.	S-8
S8	ROESY NMR (TFA-*d*, 700 MHz) spectrum of **1**.	S-9
S9	^{1}H NMR (DMSO-d_6, 700 MHz) spectrum of **1**.	S-10
S10	^{13}C NMR (DMSO-d_6, 175 MHz) spectrum of **1**.	S-11
S11	Edited HSQC NMR (DMSO-d_6, 700/175 MHz) spectrum of **1**.	S-12
S12	HSQC-TOCSY NMR (DMSO-d_6, 700/175 MHz) spectrum of **1**	S-13
S13	HMBC NMR (DMSO-d_6, 700/175 MHz) spectrum of **1**.	S-14
S14	CASE outcomes obtained the Molecular Connectivity Diagram resulting from TFA-*d* analyses of **1**. All eight ranked structures are depicted.	S-15
S15	Molecular Connectivity Diagram adapted to DMSO-d_6 NMR signals of **1**.	S-16
S16	Experimental and DFT-calculated ^{1}H and ^{13}C chemical shifts (ppm), RMSD, max_dev and R^2 for three top-ranked CASE-deduced candidates for **1**, along with their 3D rendering, cartesian coordinates and free energies.	S-17

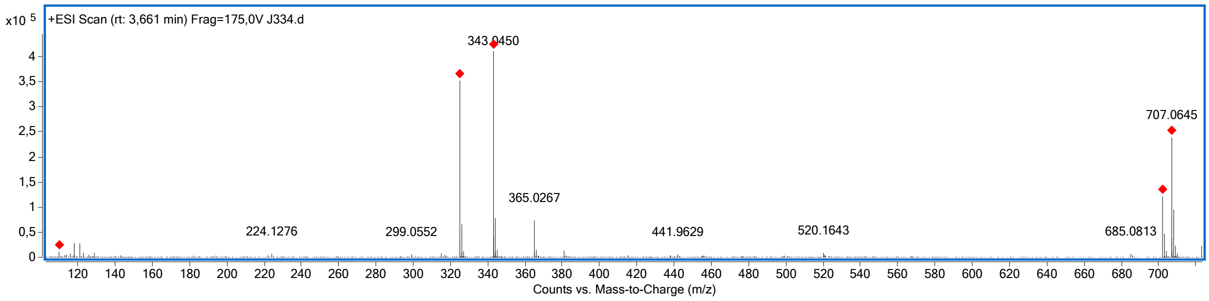

Figure S1. MS spectrum of **1**.

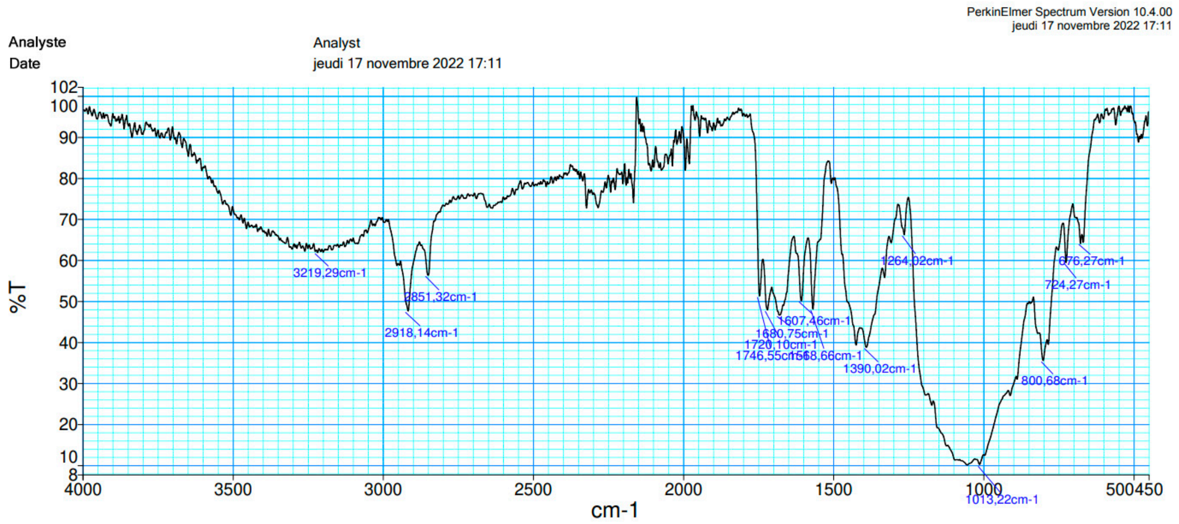

Figure S2. IR spectrum of **1**.

Figure S3. ^{1}H NMR (TFA-*d*, 700 MHz) spectrum of **1**.

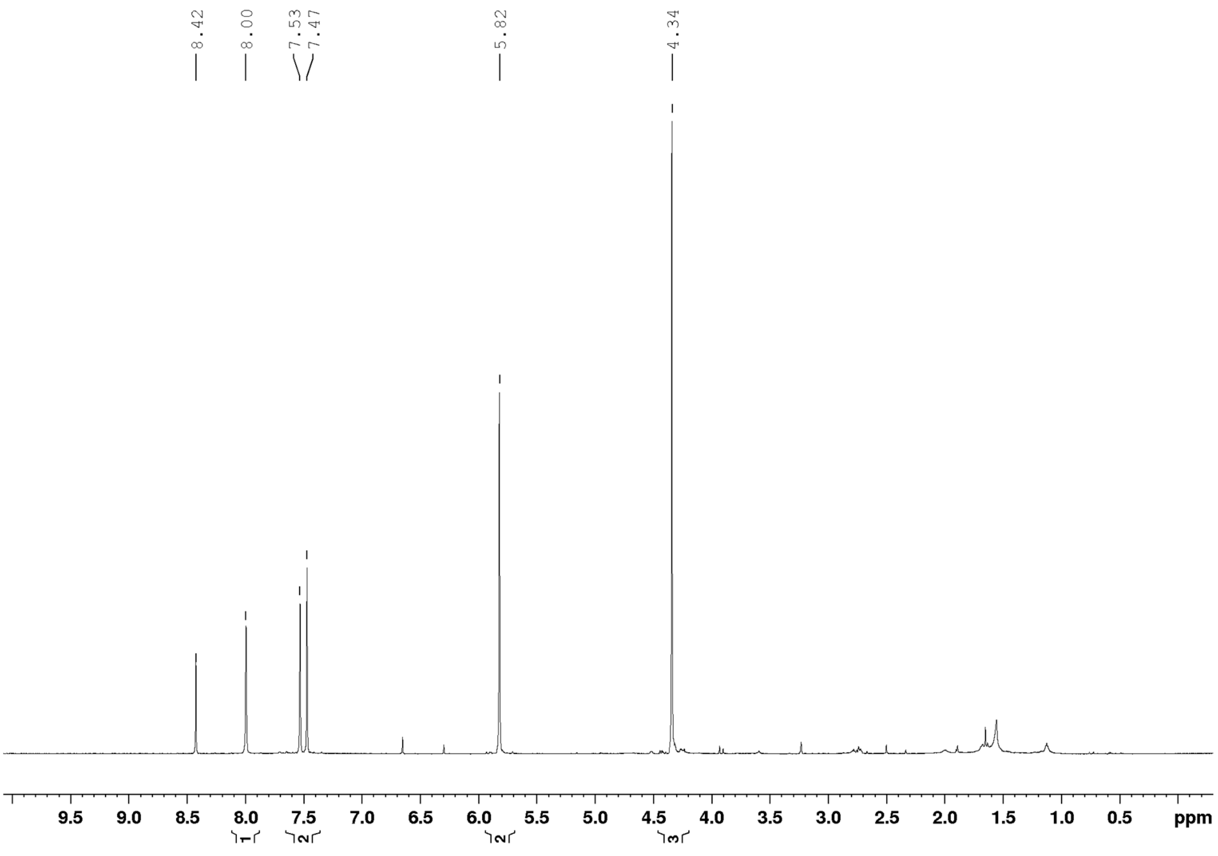

Figure S4. ^{13}C NMR (TFA-*d*, 175 MHz) spectrum of **1**.

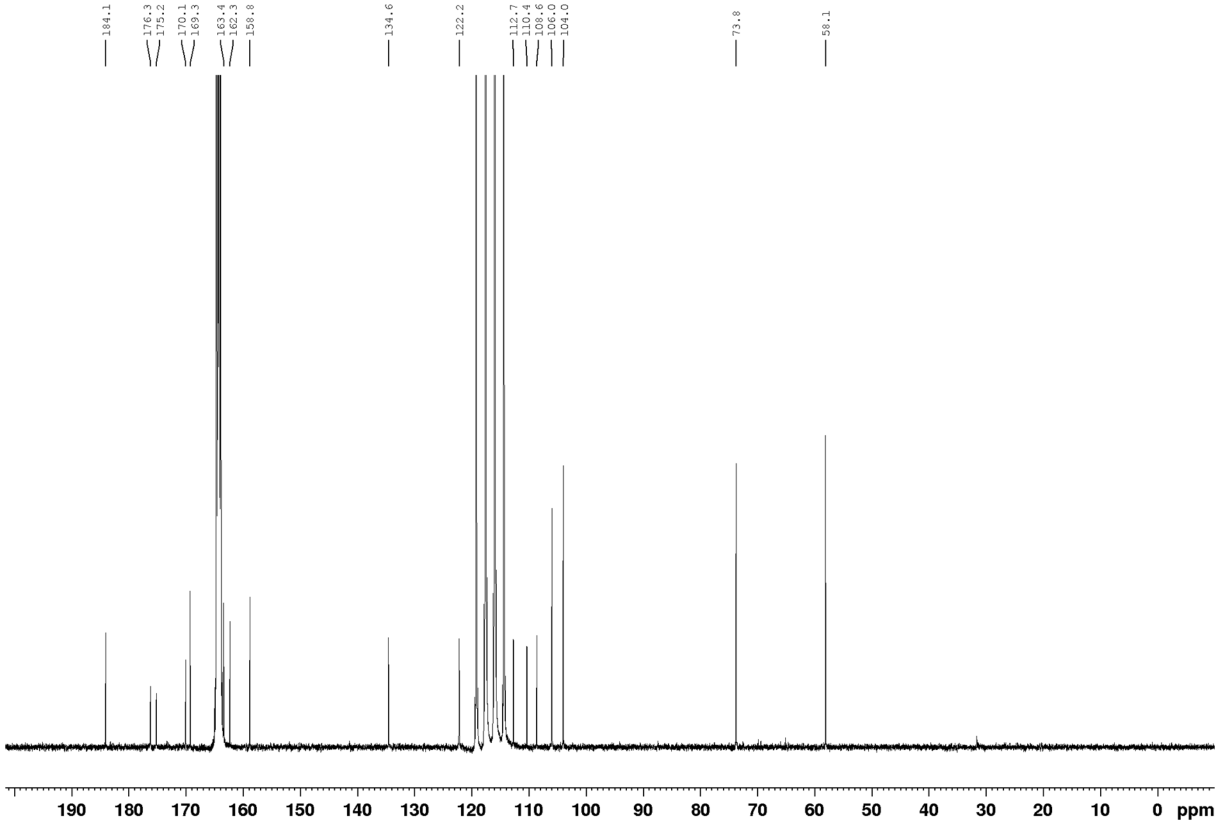

Figure S5. COSY NMR (TFA-*d*, 700 MHz) spectrum of **1**.

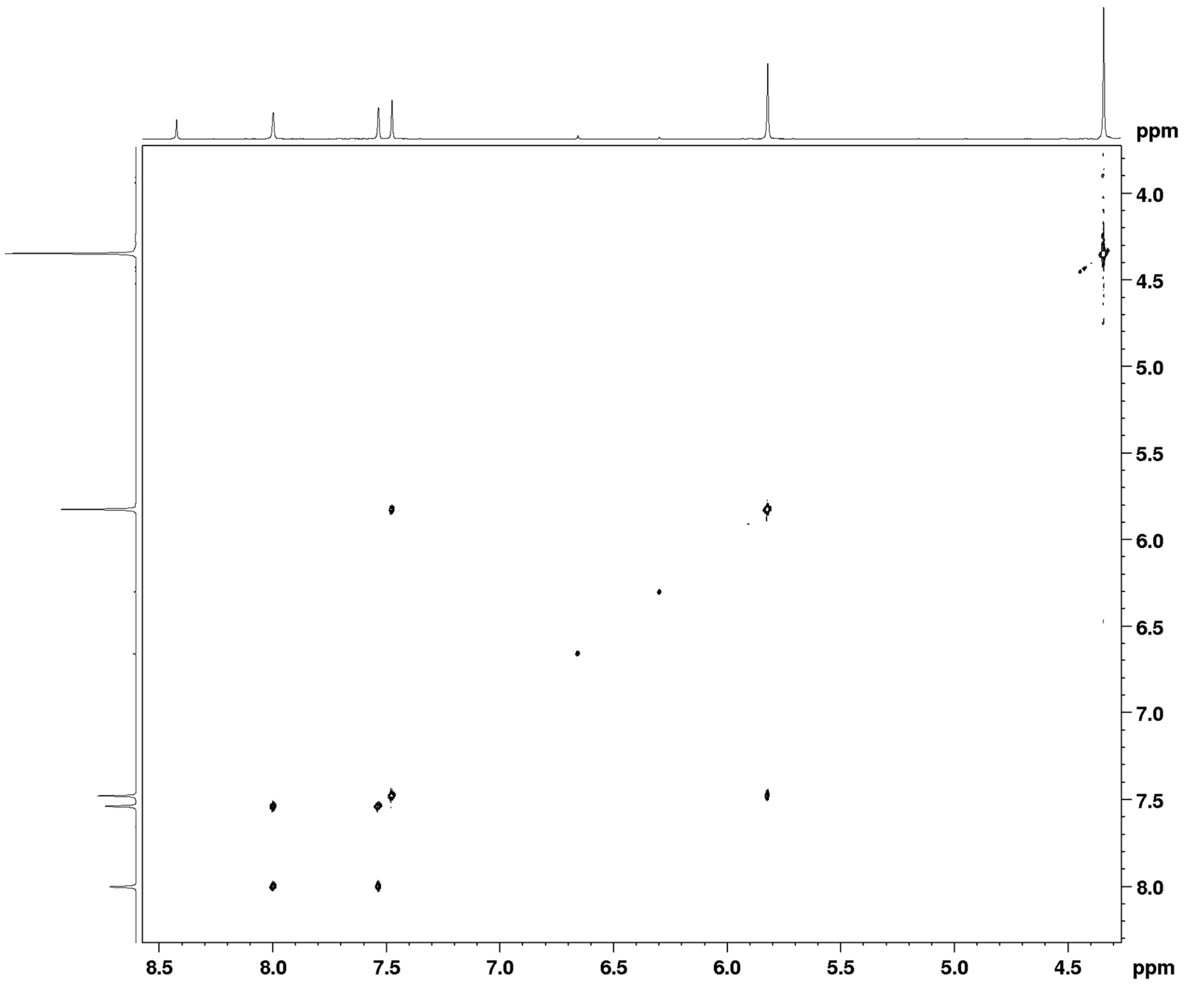

Figure S6. Edited HSQC NMR (TFA-*d*, 700/175 MHz) spectrum of **1**.

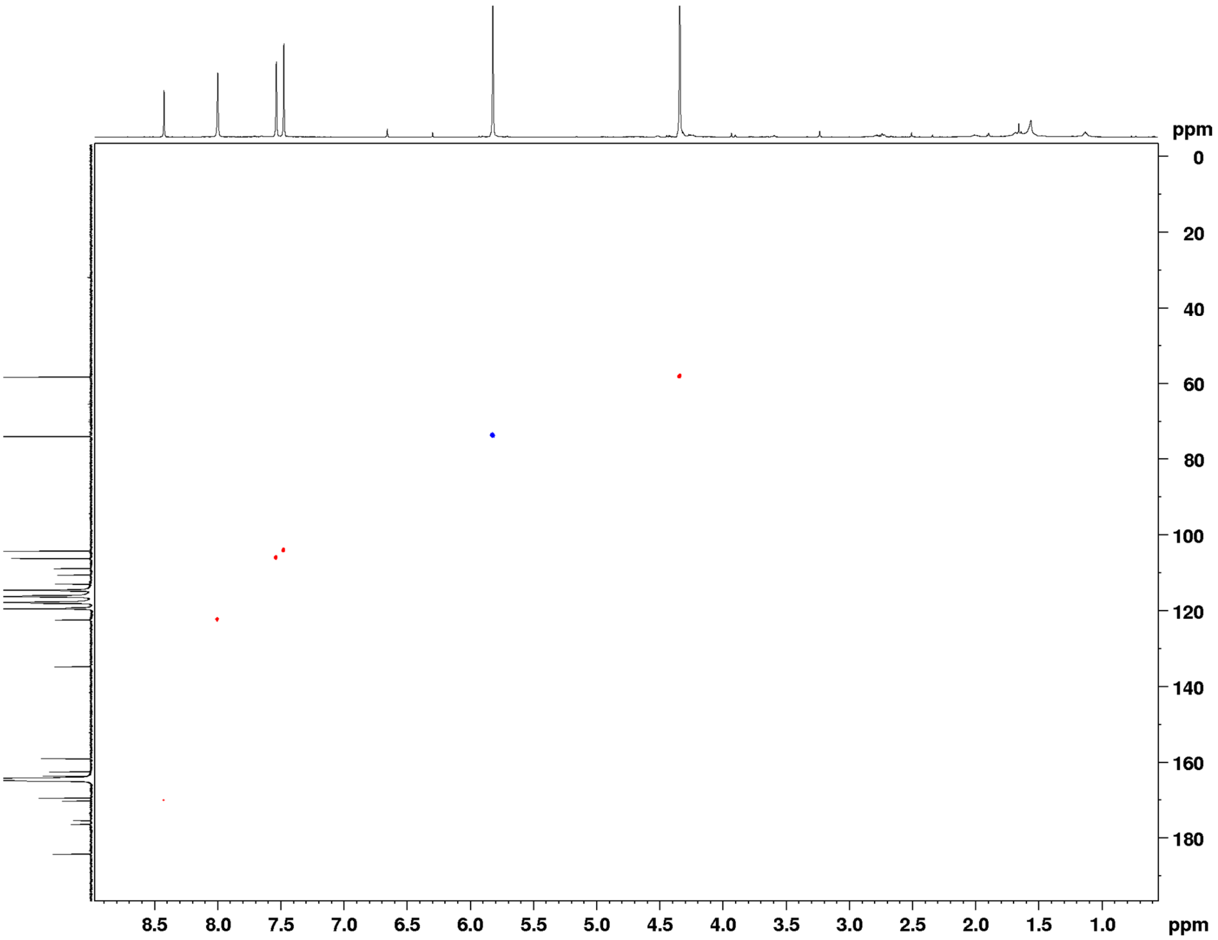

Figure S7. HMBC NMR (TFA-*d*, 700/175 MHz) spectrum of **1**.

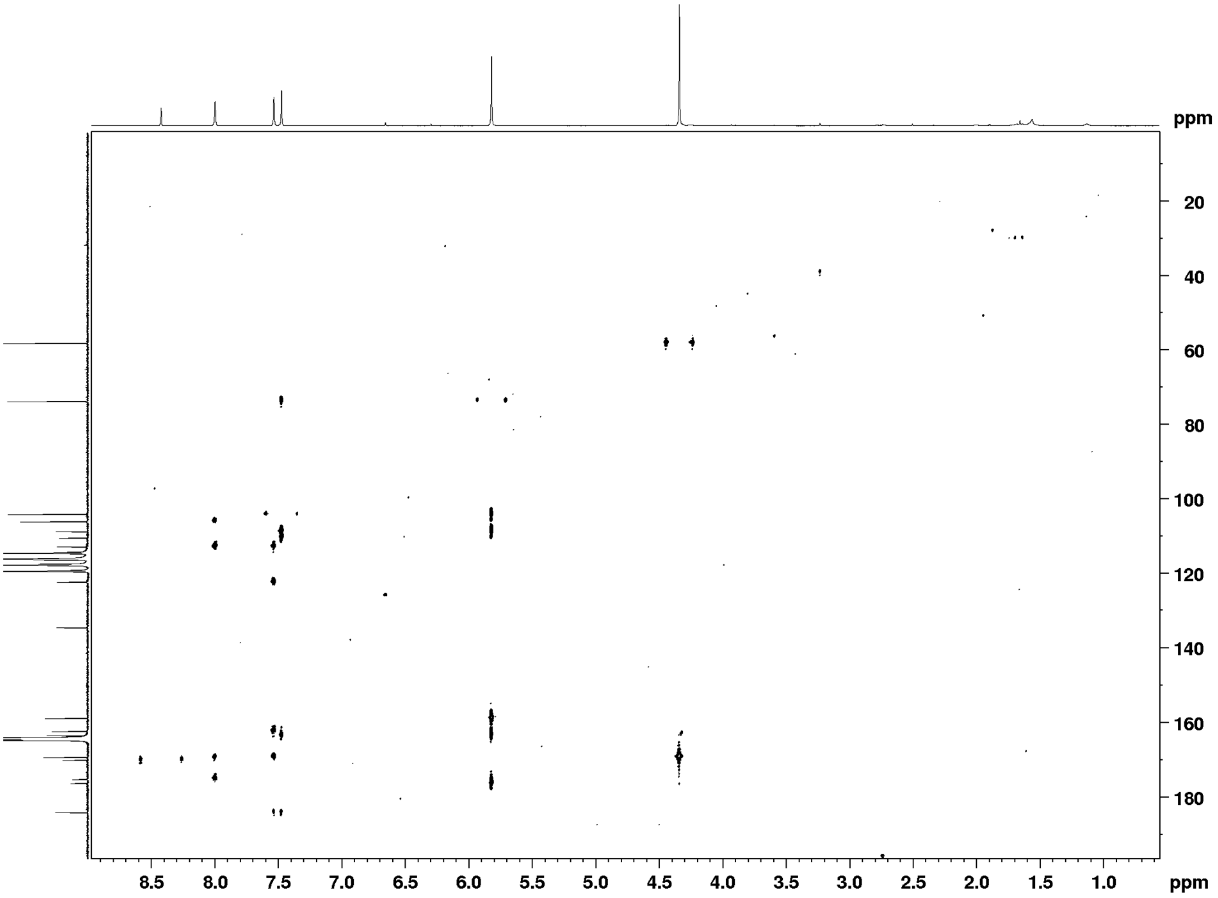

Figure S8. ROESY NMR (TFA-*d*, 700 MHz) spectrum of **1**.

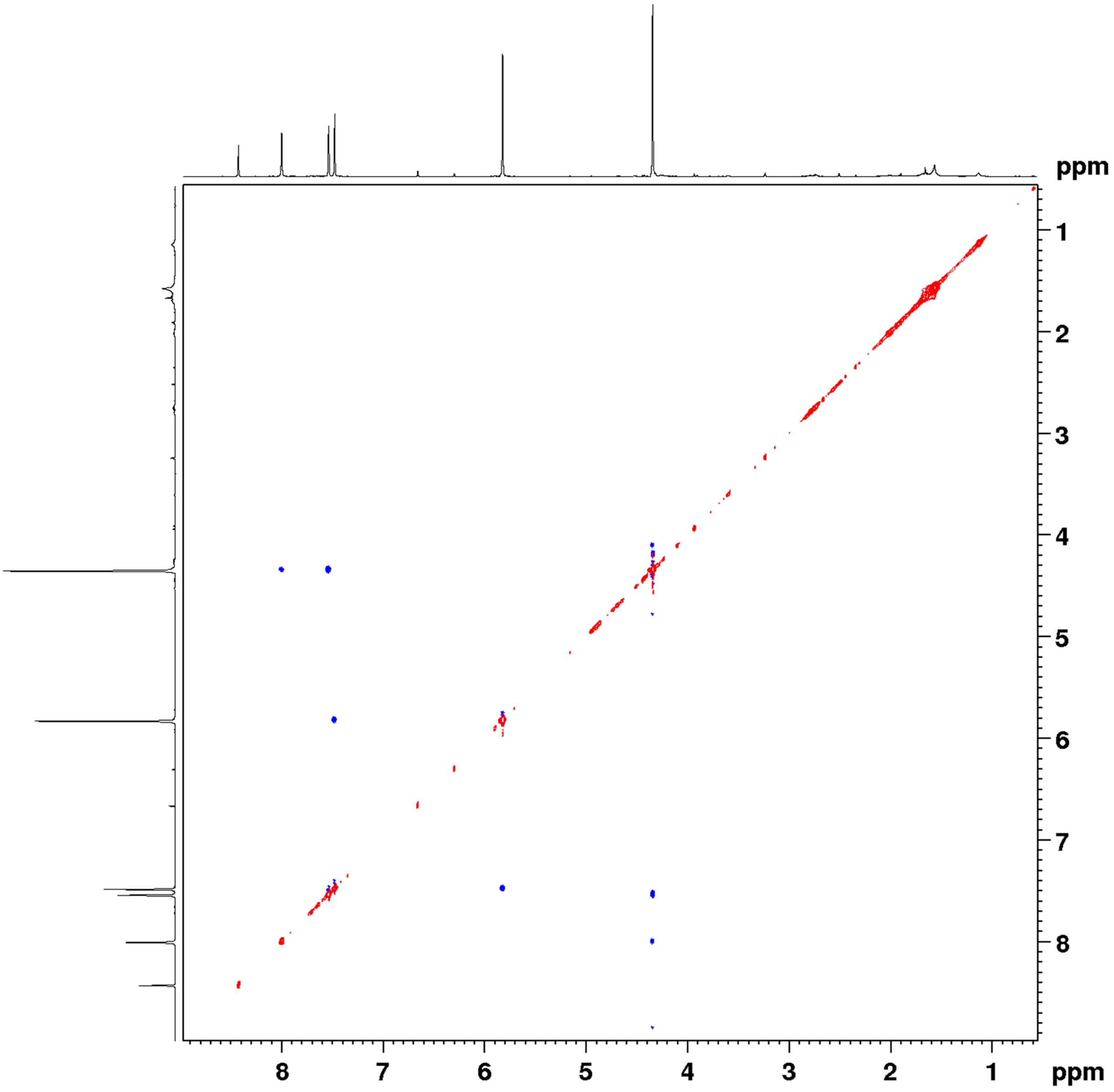

Figure S9. ^{1}H NMR (DMSO-d_6, 700 MHz) spectrum of **1**.

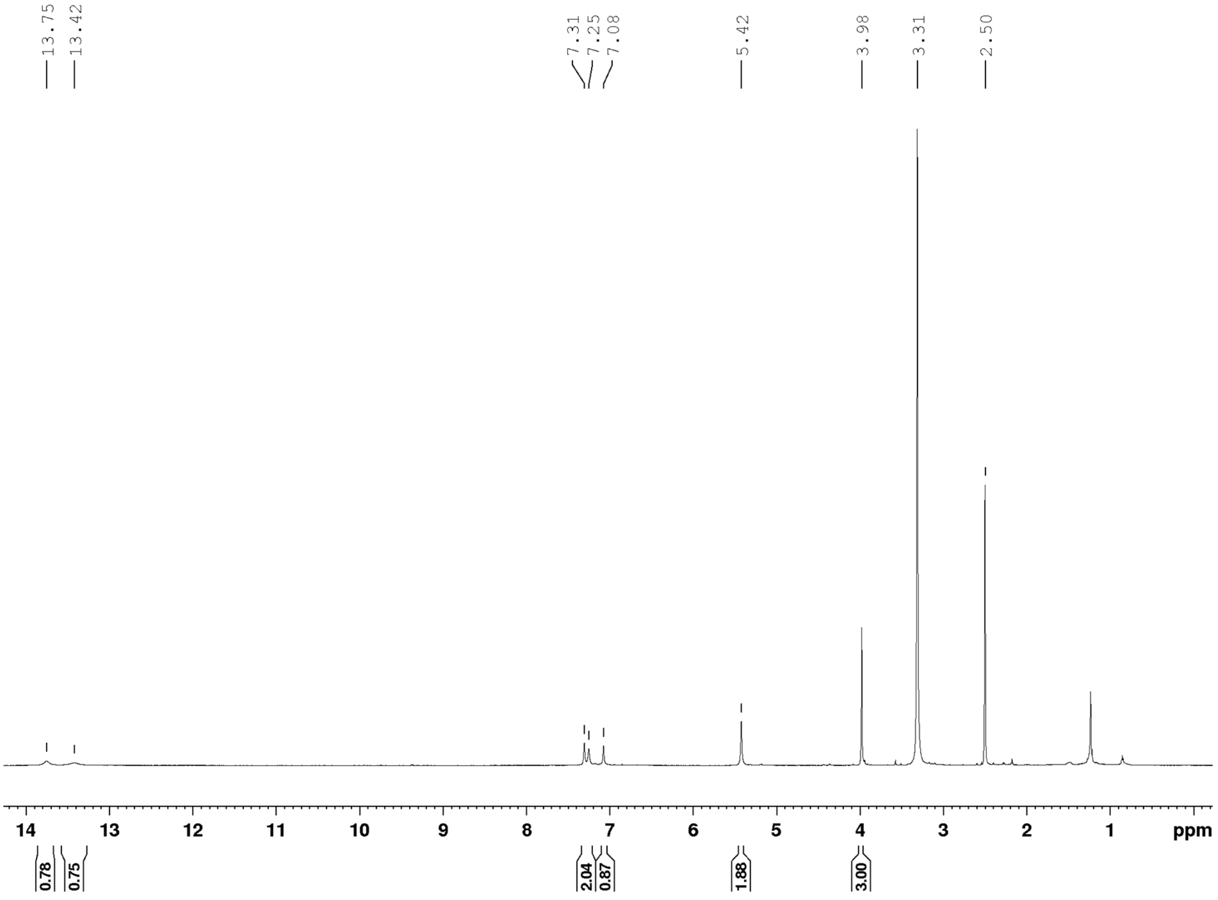

Figure S10. ^{13}C NMR (DMSO-d_6, 175 MHz) spectrum of **1**.

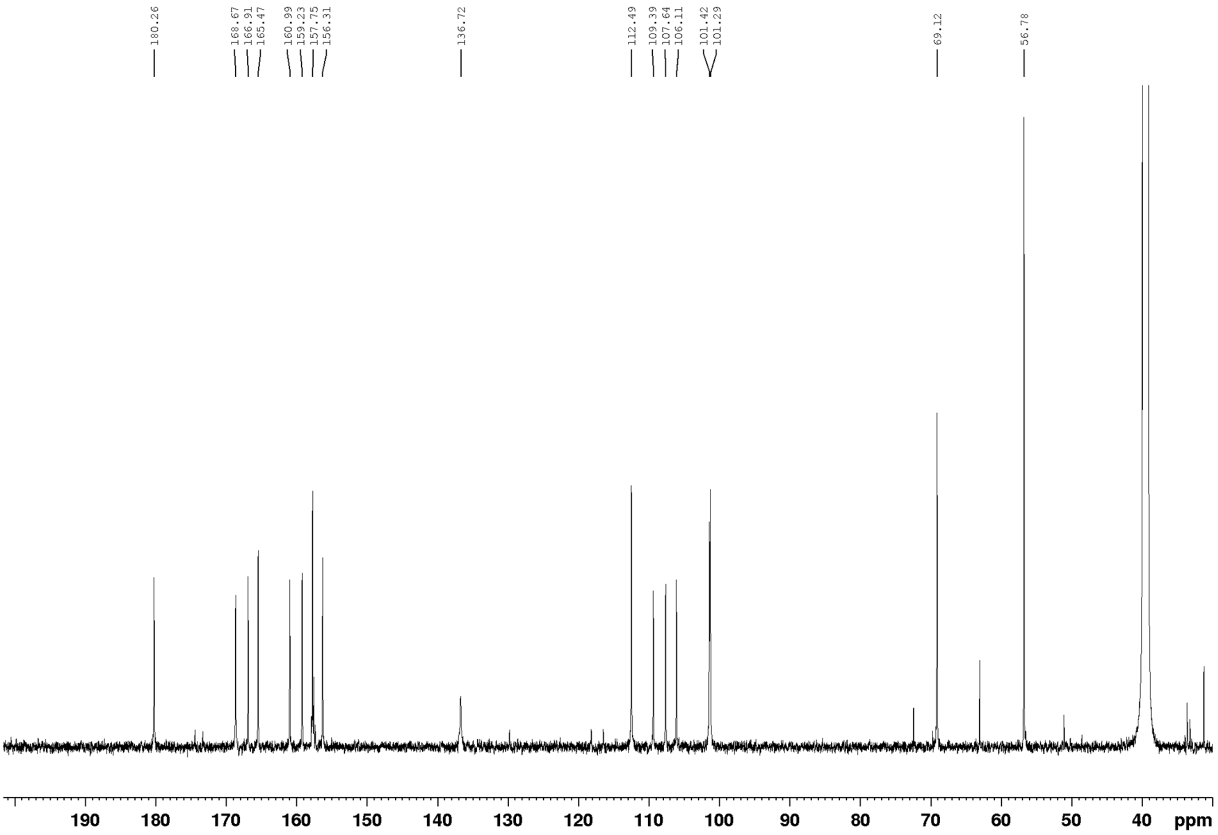

Figure S11. Edited HSQC NMR (DMSO-d_6, 700/175 MHz) spectrum of **1**.

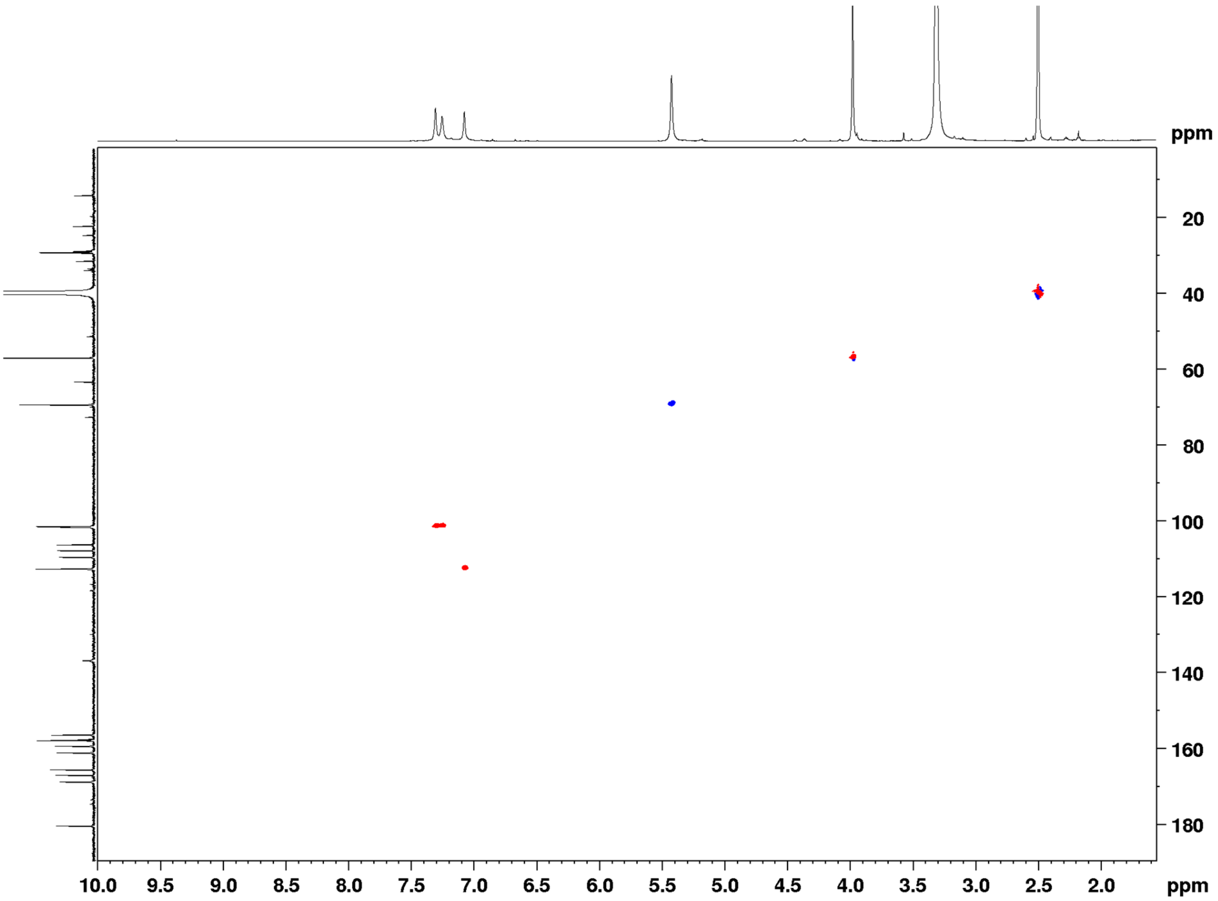

Figure S12. HSQC-TOCSY NMR (DMSO-d_6, 700/175 MHz) spectrum of **1**.

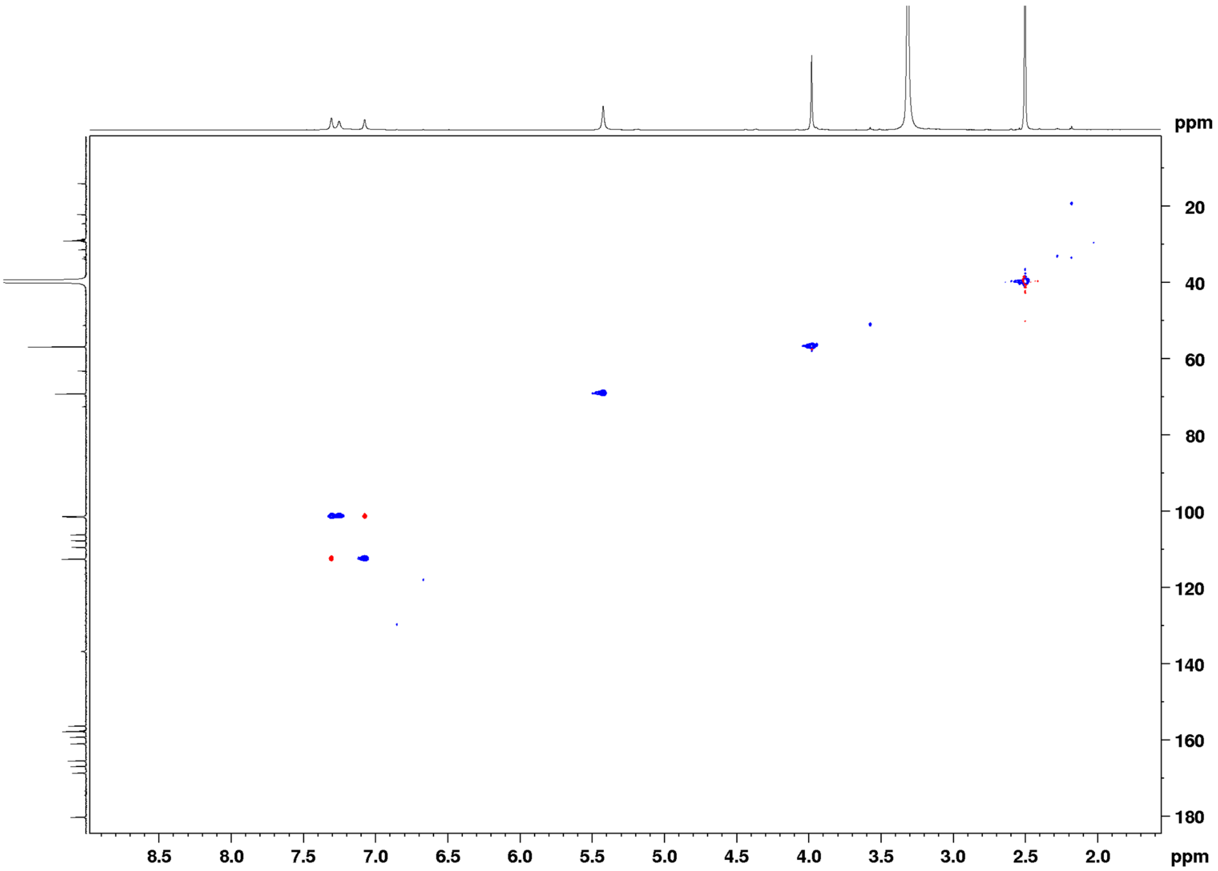

Figure S13. HMBC NMR (DMSO-d_6, 700/175 MHz) spectrum of **1**.

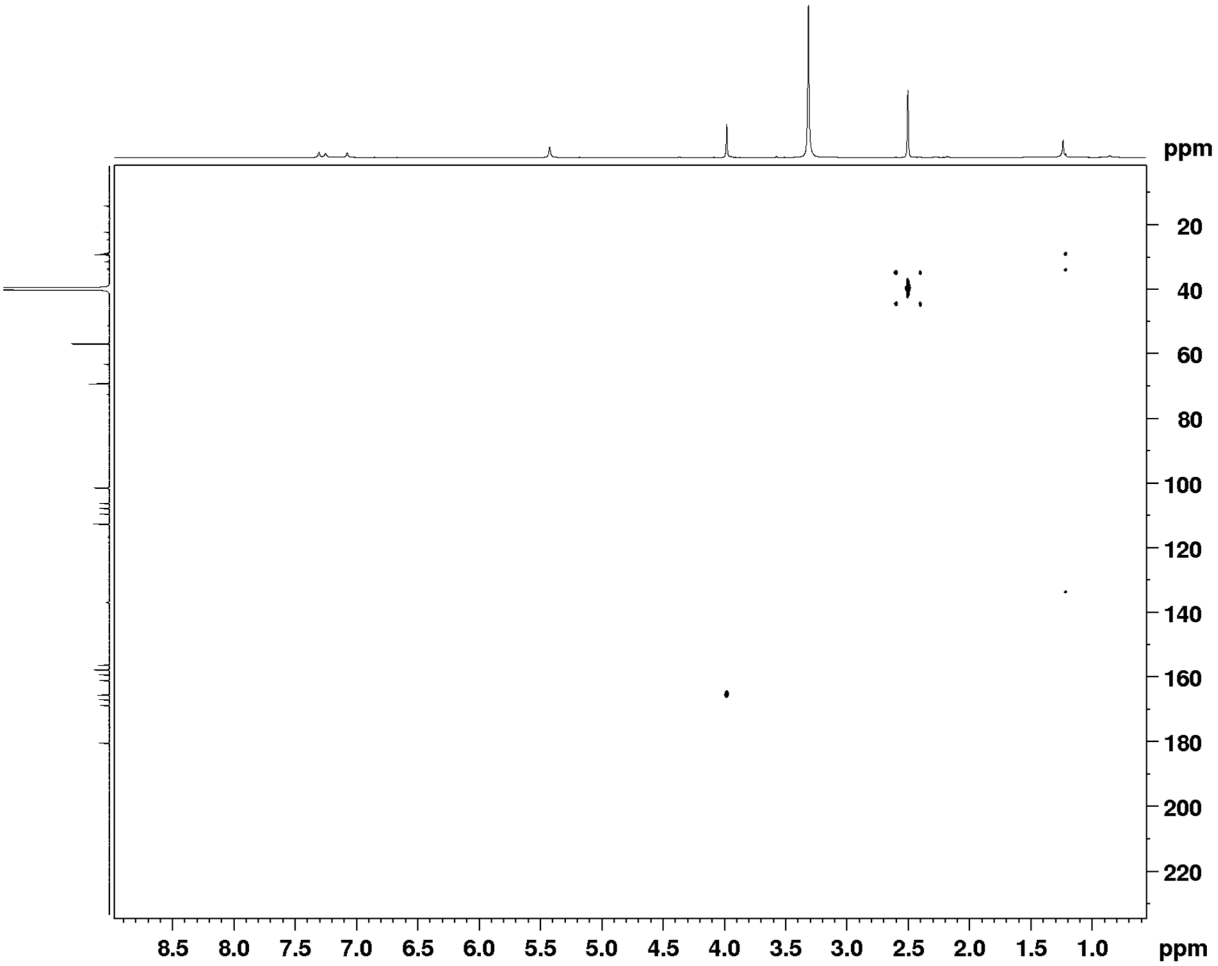

Figure S14. Eight top-ranked candidate structures listed by order of increasing spectroscopic discrepancies. Average deviations are reported according to the prediction method : d_A, HOSE-code ; d_N,neural network and d_I, incremental network along with the maximal ^{13}C chemical shift deviation for each prediction method. The color code refers to the accuracy of the ^{13}C NMR chemical shift prediction as follows: green = difference between theoretical and experimental < 3 ppm, yellow = difference comprised between 3 and 15 ppm and red = difference over 15 ppm. The rather low quality of similarity between experimental and theoretical ^{13}C chemical shift values was tentatively related to the use of TFA-*d* as the NMR solvent, known to exert a considerable influence on NMR chemical shift values.

1<br>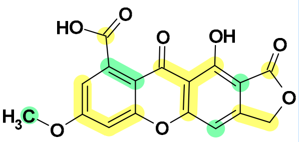	2<br>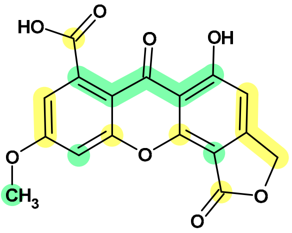	3<br>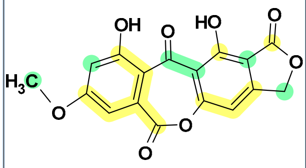	4<br>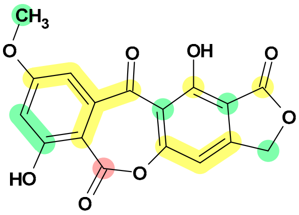
$d_A(^{13}C)$: 4.169 (v.14.56)<br>$d_N(^{13}C)$: 3.765<br>$d_I(^{13}C)$: 4.113<br>max_$d_A(^{13}C)$: 10.650<br>max_$d_N(^{13}C)$: 10.792	$d_A(^{13}C)$: 4.214 (v.14.56)<br>$d_N(^{13}C)$: 4.172<br>$d_I(^{13}C)$: 5.224<br>max_$d_A(^{13}C)$: 12.470<br>max_$d_N(^{13}C)$: 13.697	$d_A(^{13}C)$: 4.572 (v.14.56)<br>$d_N(^{13}C)$: 4.303<br>$d_I(^{13}C)$: 5.266<br>max_$d_A(^{13}C)$: 12.620<br>max_$d_N(^{13}C)$: 14.613	$d_A(^{13}C)$: 5.349 (v.14.56)<br>$d_N(^{13}C)$: 5.861<br>$d_I(^{13}C)$: 5.620<br>max_$d_A(^{13}C)$: 16.080<br>max_$d_N(^{13}C)$: 15.308
5<br>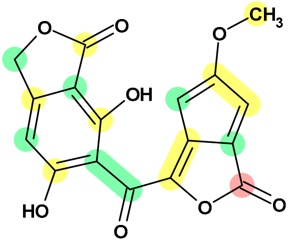	6<br>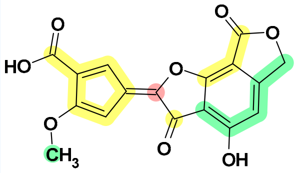	7<br>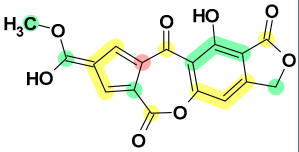	8<br>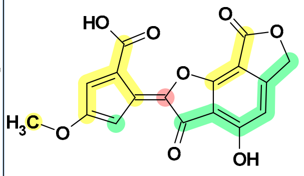
$d_A(^{13}C)$: 6.027 (v.14.56)<br>$d_N(^{13}C)$: 5.700<br>$d_I(^{13}C)$: 5.798<br>max_$d_A(^{13}C)$: 20.670<br>max_$d_N(^{13}C)$: 20.773	$d_A(^{13}C)$: 6.227 (v.14.56)<br>$d_N(^{13}C)$: 5.796<br>$d_I(^{13}C)$: 5.443<br>max_$d_A(^{13}C)$: 15.360<br>max_$d_N(^{13}C)$: 15.636	$d_A(^{13}C)$: 6.378 (v.14.56)<br>$d_N(^{13}C)$: 8.085<br>$d_I(^{13}C)$: 5.542<br>max_$d_A(^{13}C)$: 30.830<br>max_$d_N(^{13}C)$: 29.671	$d_A(^{13}C)$: 6.471 (v.14.56)<br>$d_N(^{13}C)$: 6.084<br>$d_I(^{13}C)$: 5.879<br>max_$d_A(^{13}C)$: 15.250<br>max_$d_N(^{13}C)$: 15.525

Figure S15. Molecular Connectivity Diagram (MCD) obtained from the NMR data acquired in DMSO-d_6. Carbons hybridized as sp^3 and sp^2 are respectively written in navy blue and violet. The admissibility of a neighbor heteroatom is indicated by the labels ob (obligatory) and fb (forbidden). HMBC connectivities are indicated by green arrows. Violet arrows refer to nonstandard correlations (length n > 3, for $^nJ_{HH,CH}$). These correlations were set for the weak HMBC signals. All HMBC and COSY connectivities (green and blue arrows correspondently) are shown below MCD, appearing on the structure of Ominoxanthone (**1**).

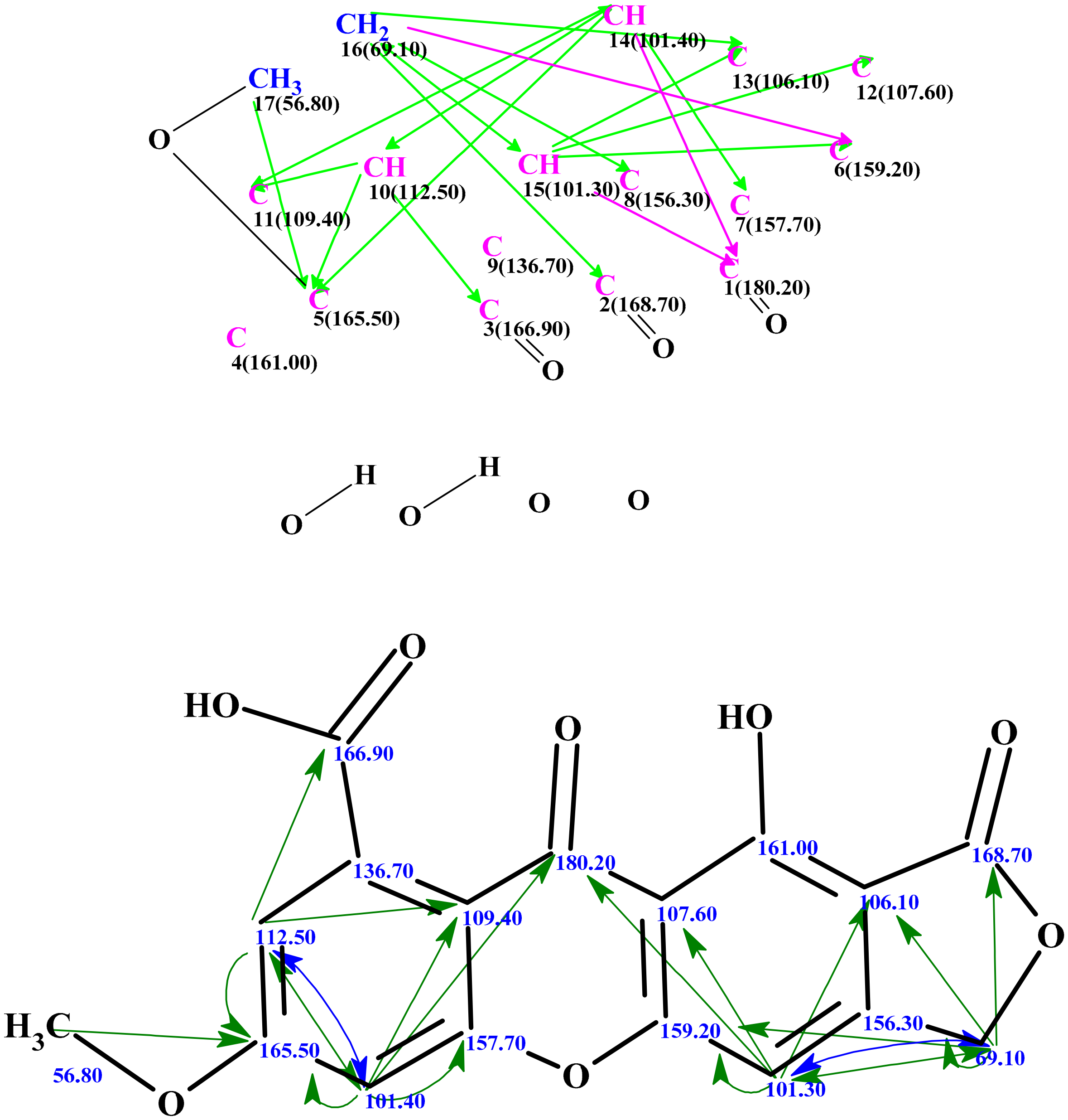

Figure S16. Experimental and DFT-calculated ^{1}H and ^{13}C chemical shifts (ppm), RMSD, max_dev and R^2 for three top-ranked CASE-deduced candidates for **1**, along with their 3D rendering, cartesian coordinates and free energies.

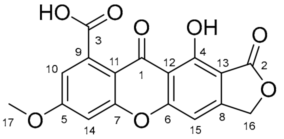

1

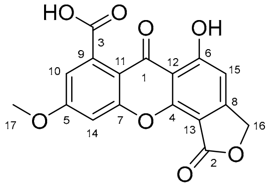

2

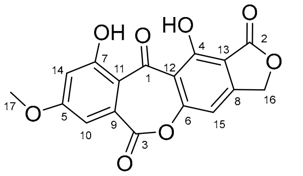

3

DFT analysis of ^{1}H NMR chemical shifts in compounds **1-3**

Protons	Exp. (DMSO)	**1**	**2**	**3**
10	7.07	6.93	6.92	6.77
14	7.30	6.94	7.03	7.40
15	7.25	6.87	6.73	6.73
16	5.42	5.15	5.14	5.07
17	3.98	3.82	3.84	3.80
RMSD, ppm		0.32	0.34	0.36
Max_dev, ppm		0.37	0.52	0.52
R^2		0.998	0.995	0.988

DFT analysis of ^{13}C NMR chemical shifts in compounds **1-3**

Carbons	Exp. (DMSO)	**1**	**2**	**3**
1	180.25	177.70	176.91	191.89
2	168.67	166.94	166.62	167.46
3	166.90	169.15	169.18	162.13
4	160.99	161.46	151.87	161.23
5	165.47	164.28	164.21	163.65
6	159.23	158.10	167.07	154.57
7	157.74	156.47	155.91	163.10
8	156.31	156.89	157.33	157.22
9	136.72	137.15	136.96	128.13
10	112.49	115.09	114.93	109.95
11	109.39	109.32	109.57	114.74
12	107.64	107.02	107.32	115.48
13	106.10	105.27	102.44	108.32
14	101.42	99.28	99.24	110.08
15	101.29	99.43	103.36	103.88
16	69.12	68.39	68.68	68.48
17	56.78	53.95	54.17	53.75
RMSD, ppm		1.66	3.59	5.48
Max_dev, ppm		2.83	9.11	11.64
R^2		0.9992	0.9956	0.9898

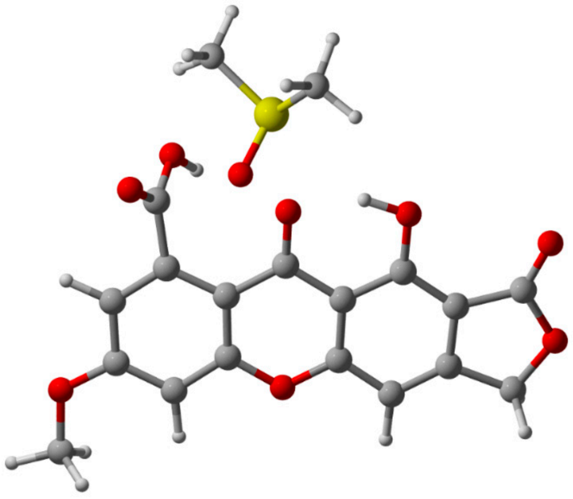

1

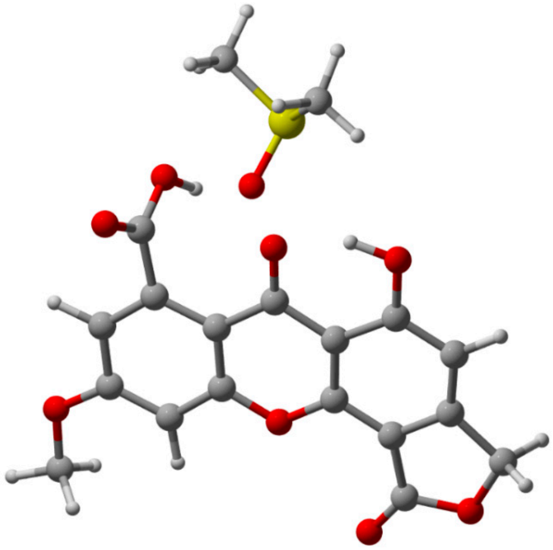

2

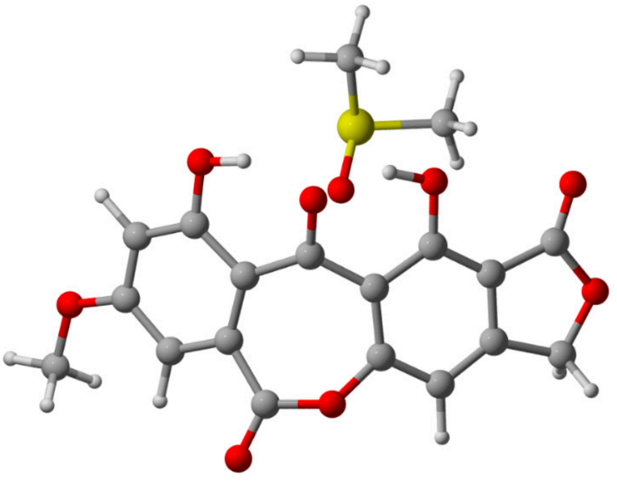

3

Structure **1**
G=-1808.677532 Hartree

C	-2.53581600	-0.27420400	0.84951100
C	-3.64347400	-1.03969900	0.53533700
C	-3.50054600	-2.27674400	-0.12941100
C	-2.23498600	-2.74717000	-0.47753700
C	-1.12536100	-1.96917400	-0.14100500
C	-1.23064000	-0.73855100	0.53012900
O	0.08244600	-2.49955400	-0.51161000
C	-0.01091900	-0.02952600	0.90002300
C	-2.81962600	1.05449400	1.53001100
O	-4.65544700	-2.93158400	-0.39070000
C	-4.60099300	-4.19406800	-1.05470500
O	-3.32192100	1.09800600	2.63181500
O	-2.61795600	2.15195900	0.79660600
H	-2.11613300	1.99306800	-0.05760200
O	-0.02236500	1.02332900	1.57694300
C	1.24420700	-0.61714100	0.44462400
C	1.24622000	-1.83730800	-0.26066100
C	2.48291800	0.04683200	0.70017000
C	3.65547500	-0.55035000	0.21373400
C	3.60135500	-1.76351800	-0.47679200
C	2.41737100	-2.43990900	-0.73039300
C	5.06253900	-0.10553300	0.29653000
O	5.55795800	0.87656100	0.78659600
O	5.82995600	-1.06894200	-0.34151200
C	5.01287300	-2.13477100	-0.84890400
O	2.51283500	1.19441500	1.36837400
H	1.56635800	1.38639400	1.63880300
O	-1.22765800	2.15710800	-1.43609000
S	-0.67269100	3.57203000	-1.69811500
C	0.29508800	4.04003400	-0.21899700
C	-2.07401700	4.72187300	-1.44889300
H	-4.64056000	-0.70190500	0.79418600
H	-2.07430600	-3.68496700	-0.99235700
H	-5.63626700	-4.52165700	-1.14698300
H	-4.15668700	-4.09595300	-2.05199100
H	-4.03584700	-4.92502400	-0.46495400
H	2.36906100	-3.38227600	-1.26408300
H	5.15992400	-2.20402300	-1.93260000
H	5.33628000	-3.07515900	-0.38843800
H	1.19389700	3.42123700	-0.21077000
H	-0.29898500	3.85305100	0.67844600
H	0.57381000	5.09395700	-0.30362100
H	-2.79476300	4.51695800	-2.24266700
H	-2.52663000	4.54118100	-0.47090000
H	-1.70889200	5.74885000	-1.53462800

Structure **2**
G=-1808.680181 Hartree

C	1.81011100	1.41875100	0.86670300
C	2.08030100	2.73176100	0.53025800
C	1.08440500	3.54962300	-0.04879100
C	-0.19326100	3.04410300	-0.28259500
C	-0.45381400	1.72118300	0.07719100
C	0.51173400	0.87768800	0.65655500
O	-1.73300300	1.30432700	-0.17472500
C	0.12944600	-0.47436400	1.04287200

C	2.96411600	0.63145800	1.46302600
O	1.46966900	4.81168400	-0.34351300
C	0.51956400	5.70633800	-0.92583400
O	3.40582900	0.89594700	2.55960800
O	3.54255500	-0.26724400	0.66114200
H	3.03324400	-0.45199400	-0.18333200
O	0.91827500	-1.26549300	1.61046100
C	-1.24363500	-0.86735000	0.74134200
C	-2.12156500	0.04667300	0.13392500
C	-1.71046000	-2.18350200	1.05954800
C	-3.02585100	-2.56918000	0.76454800
C	-3.85259300	-1.63083700	0.16274400
C	-3.43430900	-0.33853500	-0.15689200
O	-5.62985100	-0.46800800	-0.82082100
C	-5.29340400	-1.74911300	-0.26069800
O	-0.88682100	-3.06042100	1.64024400
H	-0.01848900	-2.57939500	1.79140900
C	-4.55148100	0.39814300	-0.77612500
O	-4.62225200	1.52721700	-1.19506100
C	4.85449800	-2.36376800	-1.74694800
S	3.03655500	-2.53102000	-1.87330600
O	2.45419800	-1.13735900	-1.56666700
C	2.77946600	-3.54633900	-0.37354400
H	3.06176800	3.15779100	0.70576500
H	-0.99255400	3.62468200	-0.72360900
H	1.05183000	6.64667100	-1.06697100
H	0.16950800	5.33212600	-1.89448900
H	-0.33386000	5.86309800	-0.25659600
H	-3.35421200	-3.57157300	1.01704200
H	-5.44763500	-2.51637500	-1.02738300
H	-5.96602500	-1.94977100	0.58047100
H	5.16743200	-1.70838300	-2.56186600
H	5.31065100	-3.35009200	-1.86748500
H	5.11482900	-1.92023800	-0.78286200
H	3.30806300	-4.49539600	-0.49822200
H	3.13552400	-2.99902700	0.50219100
H	1.70664300	-3.72738900	-0.29022800

Structure **3**
G=-1808.648393 Hartree

C	-2.55218400	1.10971400	1.11640000
C	-3.75169400	1.31277100	0.43166400
C	-4.27706400	0.31535800	-0.38174400
C	-3.61858400	-0.92272900	-0.47927100
C	-2.40260300	-1.12606800	0.16661600
C	-1.79629300	-0.10402100	0.95122200
C	-0.43588900	-0.11781500	1.49132000
O	-2.14322800	2.11992100	1.89483500
O	-5.44077600	0.60369100	-1.00922900
C	-6.04342600	-0.38298700	-1.85039100
O	-0.12588700	0.72953200	2.38309500
C	0.66112500	-0.91273200	0.91644800
C	0.49262800	-2.15240100	0.26717100
C	1.97678700	-0.33940300	0.93408000
C	2.98889300	-0.97273500	0.19369200
C	2.75800800	-2.18270000	-0.44754800
C	1.52250800	-2.81281100	-0.40337800
C	4.38286800	-0.55457100	-0.05734100

O	4.98322600	0.44277400	0.26004600
O	4.97339900	-1.54704400	-0.82008900
C	4.04282600	-2.60522800	-1.10636500
O	2.27125500	0.76483700	1.61084100
H	1.45399100	1.00563200	2.12130000
O	-0.65636800	-2.90339800	0.37459600
C	-1.95494500	-2.55216100	0.11417900
O	-2.71773500	-3.46314000	-0.10933500
H	-1.30381300	1.82292800	2.33210900
C	1.17966600	4.05391100	0.17225000
S	0.99499900	3.05372600	-1.35399400
O	0.41505200	1.70589100	-0.93079800
C	2.78363900	2.79670800	-1.66125900
H	-4.27220100	2.25567300	0.55142100
H	-4.05539800	-1.75664500	-1.00972200
H	-6.93856600	0.08886400	-2.25509000
H	-5.37367400	-0.66477400	-2.67032100
H	-6.32544200	-1.27284800	-1.27693200
H	1.32805800	-3.78272700	-0.84617600
H	3.95083000	-2.70348700	-2.19364800
H	4.43991700	-3.54187400	-0.69942800
H	0.17418000	4.21789700	0.56476800
H	1.64359300	5.01203600	-0.07848500
H	1.78646500	3.50216400	0.89388200
H	3.25105300	3.76321300	-1.87110300
H	3.24877200	2.31581900	-0.79695000
H	2.86316200	2.15341700	-2.53988800
